# Emergency contraceptive knowledge and associated factors among abortion experienced reproductive age women in Ethiopia: a multilevel analysis using EDHS 2016 data

**DOI:** 10.1186/s12884-023-06091-6

**Published:** 2023-11-09

**Authors:** Tadele Biresaw Belachew, Wubshet Debebe Negash, Daniel Gashaneh Belay, Fantu Mamo Aragaw, Melaku Hunie Asratie, Desale Bihonegn Asmamaw

**Affiliations:** 1 Department of Health Systems and Policy, Institute of Public Health, College of Medicine and Health Sciences, University of Gondar, Gondar, Ethiopia; 2 Department of Human Anatomy, College of Medicine and Health Sciences, University of Gondar, Gondar, Ethiopia; 3 Department of Epidemiology and Biostatistics, Institute of Public Health, College of Medicine and Health Sciences, University of Gondar, Gondar, Ethiopia; 4 Department of Women’s and Family health, School of Midwifery, College of Medicine and Health Sciences, University of Gondar, Gondar, Ethiopia; 5 Department of Reproductive Health, Institute of Public Health, College of Medicine and Health Sciences, University of Gondar, Gondar, Ethiopia

**Keywords:** Emergency contraceptive, Knowledge, Multilevel, Ethiopia

## Abstract

**Background:**

Emergency contraceptives (EC) are used to avoid unintended pregnancy, hence avoiding its incidence and its effects. In Ethiopia, emergency contraception is commonly accessible, especially in the big cities. However, there is virtually little understanding of or awareness of EC and Ethiopia has a high abortion rate. Therefore this study was aimed to assess the magnitude and associated factors for emergency contraceptive knowledge in Ethiopia.

**Methods:**

The study was based on secondary data analysis of the Ethiopian Demographic and Health Survey 2016 data. A total weighted sample of 1236 reproductive age women was included. A multilevel mixed-effect binary logistic regression model was fitted to identify the significant associated factors of emergency contraceptive knowledge. Statistical significance was determined using Adjusted Odds Ratio (AOR) with 95% confidence interval.

**Results:**

Overall magnitude of emergency contraceptive knowledge was observed to be 17.19% (95% CI: 15.18, 19.40) with intra-class correlation (ICC) 57% and median odds ratio (MOR) 6.4 in the null model. Women’s age 25–34 (AOR = 2.6; 95% CI: 1.2, 5.5), and 35–49 (AOR = 1.5; 95% CI: 1.06, 3.3), secondary and above educational level (AOR = 3.41; 95% CI: 2.19, 4.88), media exposure (AOR = 2.97; 95% CI: 1.56, 5.64), Being in metropolitan region (AOR = 2.68; 95% CI: 1.46, 4.74), and women being in urban area (AOR = 3.19; 95% CI: 1.20, 5.23) were associated with emergency contraceptive knowledge.

**Conclusion:**

Emergency contraceptive knowledge in this study was low. Women age, educational level, media exposure, residency, and region were significantly associated with emergency contraceptive knowledge. Therefore, to enhance understanding and use of ECs in the current Ethiopian setting, it is imperative to ensure exposure to EC information, particularly in rural regions.

## Background

Emergency contraceptives (EC) are the only method available to women to prevent pregnancy after unprotected sexual contact, a contraceptive failure, forgetting to take their birth control pills too late, or being forced to have sex against their will [1, 2]. EC is also known as “morning-after” or “post-coital” contraception [3]. EC is only intended for occasional or emergency use and should not be used as a regular method of contraception [4, 5]. previously, EC was thought to be effective only for 72 h, but recent studies have confirmed that it is effective for up to 120 h [3]. The copper-releasing intrauterine device (IUD) can be safely used for EC up to 5 days after unprotected intercourse, lowering the risk of pregnancy by more than 99% [6, 7].

EC is largely underutilized worldwide, and is considered one of the best kept secrets in reproductive medicine [8]. Globally, EC is not widely used. According to reports, 9.4% of Americans use it [9], in South Africa as 4% [10] and in Iran as 5.2% [11]. Additionally, studies have shown a lack of knowledge and attitude about emergency contraception among women [12]. Every year, approximately 250 million pregnancies occur worldwide [13, 14]. One-third of these pregnancies are unintended, with 20% ending in abortion. More than one-third of the 182 million pregnancies in low-income countries are unintended; of which 19% are terminated by induced abortion. Among this 11% of induced abortions are unsafe [15].

Because of the high unmet need for family planning and the consequences, many Ethiopian women face the difficulties of abortion and unwanted child birth. As a result, Ethiopia’s Federal Ministry of Health has authorized the distribution of EC in drug stores as well as the provision of safe abortion services in medical facilities for those who require the service under certain conditions such as rape, incest, sexual violence, and so on.

Throughout Ethiopia, EC pills can be found in any drug retails in a two dose oral pills that should be taken in 12 h apart. Based on the information on the leaflet distributed with the drug, the two doses should be taken within 72 h after exposure to unprotected sex. Despite the fact that EC is easily accessible from drug stores in Ethiopia’s major cities, the national abortion rate is quite high which account the average of 49 per 1,000 [16, 17].

Abortion, even when done safely, can be painful and cause psychological and physical stress. The paradox here is that, while EC services are widely available, why would women prefer to have an abortion in the face of potential complications? There are several reasons for this, but the most important is a lack of adequate knowledge about EC among the general public, particularly women.

However, specific developing countries have studied the knowledge of emergency contraceptive method among reproductive-age women, including Ethiopia [18–21], Nigeria [22], South Africa [23], Nairobi [24] and Botswana [25]. There have been no studies that have used national EDHS representative data in Ethiopia. In addition, these previous studies failed to consider community level factors and their interaction with individual-level factors. Multilevel approaches will provide an understanding of factors affecting ECK at both the individual and community levels.

Therefore, this study aimed to assess the knowledge of emergency contraceptive and its associated factors among abortion experienced reproductive age women in Ethiopia. The findings of this study will be used to implement interventions to increase awareness and use of EC, thereby reversing the occurrence of unwanted pregnancy and its consequences.

## Methods

### Study design, period, and setting

A community-based cross-sectional survey was conducted using secondary data in the 2016 Ethiopian Demographic and Health Surveys (EDHS), which was conducted by the Central Statistical Agency (CSA) in collaboration with the Federal Ministry of Health (FMoH) and the Ethiopian Public Health Institute (EPHI), which was a national representative sample conducted from January 18 to June 27, 2016 [26]. There are nine regional states in Ethiopia (Tigray, Afar, Amhara, Oromia, Benishangul, Gambela, South Nation, Nationalities and Peoples’ Region (SNNPR), Harari, and Somali), and two administrative cities (Addis Ababa and Dire-Dawa). The data were gained from the official database of the EDHS program, www.measuredhs.com after authorization was granted via online request by explaining the purpose of our study. We extracted dependent and independent variables from the woman record (IR file). EDHS are a nationally representative household survey conducted by face-to-face interviews on a wide range of populations. Study participants were selected using a two-stage stratified sampling technique. Enumeration Areas (EAs) were randomly selected in the first stage, while households were selected in the second stage [27]. A total of weighted sample of 1236 abortion experienced reproductive age women were included.

### Variables and measurements

#### Dependent variable

The outcome variable of this study was having correct knowledge of emergency contraceptive (EC), which was recoded and dichotomized. When collecting data from the women, they were asked “Do you know emergency contraceptive?” The different responses were: “yes”, “no”. This variable coding is provided by the EDHS [28].

### Independent variables

#### Individual level variables

Age of respondents, educational status of respondents, current marital status, occupation of respondents, wealth index, media exposure, Contraceptive use, sexually activity, and religion were included.

#### Community level variables

Community level variables included residency and region were directly accessed from EDHS data sets. However, community level poverty, community level education, and community-level media exposure were constructed by aggregating individual-level characteristics at the cluster level [29]. They were categorized as high or low based on the distribution of the proportion values generated for each community after checking the distribution by using the histogram. The aggregate variable was not normally distributed and the median value was used as a cut-off point for the categorization [29, 30].

### Media exposure

Media exposure was calculated by aggregating TV watching, radio listening, and reading newspapers and woman who have exposure to either of the media sources was categorized as having media exposure and the rest considered as having no media exposure [31, 32].

### Wealth index

The variable wealth index was re-categorized as “Poor”, “Middle”, and “Rich” categories by merging poorest with poorer and richest with richer [32–35].

### Data analysis

For data analysis Stata version 16 software was used. To ensure the representativeness of the EDHS sample and obtain reliable estimations and standard errors, data were weighted (v005/1,000,000) throughout analysis.

Four models fitted: the null model with no explanatory variables, model I with individual factors, model II with community factors, and model III with both individual and community factors. To compare and assess the fitness of nested models, we used the intra class correlation coefficient (ICC), the median odds ratio (MOR), and deviation (-2LLR). Model III was the best-fitting model due to its low deviance. In multivariable analysis, variables with a p-value less than 0.2 in bivariable analysis were used. Finally, in the multivariable analysis, adjusted odds ratios with 95% confidence intervals and p-values less than 0.05 were used to identify factors of emergency contraceptive knowledge.

## Results

### Individual level factors

Half (50.40%) of the women were aged between 35 and 49 years. Regarding their educational level, 759 (61.39%) respondents were reported as had no formal education. Among the participants, 710 (57.48%) were employed. About 86.32% of the respondents were married and 899 (72.81%) of participants were sexually active. Only one-fourth (25.6%) of participants used contraceptives. With regard to their economic status, 451 (36.5%) women were from the poor wealth quintiles and 542 (43.86%) were from the rich wealth quintiles. Moreover, 694 (56.15%) respondents had no media exposure. In addition 583 (47.15%) participants were Orthodox Christian (Table 1).

**Table 1:** Individual characteristics of respondents in Ethiopia (n = 1236)

Variables	Categories	Frequency	Percentage (%)
Age of respondents	15–24	153	12.35
	25–34	460	37.25
	35–49	623	50.40
Educational status of respondents	No formal education	759	61.39
	Primary education	409	33.07
	Secondary and above	68	5.54
Occupation of respondents	Working	710	57.48
	Not working	525	42.52
Current marital status	Married	1067	86.32
	Not married	169	13.68
Wealth index	Poor	451	36.50
	Middle	243	19.64
	Rich	542	43.86
Media exposure	Yes	542	43.85
	No	694	56.15
Contraceptive use	Yes	316	25.60
	No	920	74.40
Sexual activity	Yes	899	72.81
	No	336	27.19
Religion	Orthodox Christian	583	47.15
	Muslim	392	31.70
	Protestant	243	19.68
	Others+	18	1.48

### Community level factors

Of the study participants, 717 (57.99%) reproductive age women were from communities with high proportion of community level education. Majority (65.62%) of participants had no community media exposure. Among the respondents 522 (42.22%) were under high poverty level communities. Moreover, 1081 (87.51%) participants were from large central parts of Ethiopia (Table 2).

**Table 2:** Community level characteristics of respondents in Ethiopia (n = 1236)

Variables	Categories	Frequency	Percentage (%)
Residency	Urban	259	21
	Rural	976	79
Community media exposure	Low	811	65.62
	High	425	34.38
Community level education	Low	519	42.01
	High	717	57.99
Community level poverty	Low	714	57.78
	High	522	42.22
Region	Metropolitan	102	8.25
	Large central	1081	87.51
	Small peripheral	52	4.24

### Random effects and model fitness

According to the intra-class correlation (ICC) in the null model, 57% of the overall variability of knowledge of emergency contraceptive can be attributed to cluster variability. The median odds ratio for knowledge of emergency contraceptive in the null model was 6.4, indicating that knowledge of emergency contraceptive varied between clusters. This suggests that if we randomly picked individuals from different clusters, those in the highest knowledge of emergency contraceptive cluster had a 6.4 times higher chance of having knowledge of emergency contraceptive than those in the lowest knowledge of emergency contraceptive cluster. Likewise, the proportional change in variance (PCV) increased from 24.2% in model I to 64.5% in model III (a model with individual and community variables), which indicates that the final model (Model III) best describes knowledge of emergency contraceptive variability. Deviance was also used to assess the fitness of the model. Model III was found to have the lowest deviation, so it was the best fitting model (Table 3).

**Table 3:** Model comparison and random effect analysis result in Ethiopia

Random effect	Null Model	Model I	Model II	Model III
Variance	6.2	4.7	3.5	2.2
ICC	57.21	43.67	39.23	31.05
MOR	6.4	5.6	4.8	3.8
PCV	Ref	24.2%	43.5%	64.5%
**Model fitness**				
Deviance (-2LLR)	1016	902	854	806

### Magnitude of emergency contraceptive knowledge among abortion experienced reproductive age women in Ethiopia

**Fig. 1:**
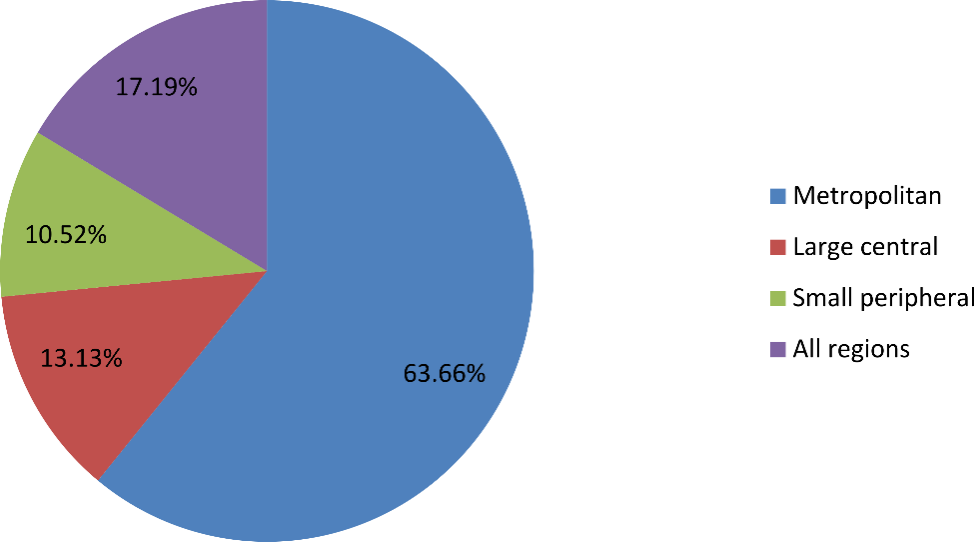
Magnitude of emergency contraceptive knowledge among abortion experienced reproductive age women in Ethiopia, 2016 EDHS data

Overall, magnitude of emergency contraceptive knowledge among abortion experienced reproductive age women in Ethiopia was 17.19% (95% CI: 15.18, 19.40) (Fig. 1).

### Factors associated with emergency contraceptive knowledge among abortion experienced reproductive age women in Ethiopia

In terms of individual level factors, the study showed that women aged 25 to 34 and 35 to 49 were more likely to have knowledge on emergency contraceptive (AOR = 2.6; 95% CI: 1.2, 5.5), (AOR = 1.5; 95% CI: 1.06,3.3) compared to 15 to 24 aged women respectively. The study found that women with a secondary and above educational level were 3.41 times more likely to have knowledge on emergency contraceptive (AOR = 3.41; 95% CI: 2.19, 4.88) than those who had no formal education. The odds of emergency contraceptive knowledge was high among reproductive age women who had media exposure (AOR = 2.97; 95% CI: 1.56, 5.64) compared with their counterparts. Regarding community level factors, women who were residing in urban area were 3.19 times to have emergency contraceptive knowledge than living in rural area (AOR = 3.19; 95% CI: 1.20, 5.23). In addition, women in metropolitan region were 2.68 more likely (AOR = 2.68; 95% CI: 1.46, 4.74) to have emergency contraceptive knowledge compared to women in small peripheral region (Table 4).

**Table 4:** Multivariable analyses for factors affecting emergency contraceptive knowledge among abortion experienced reproductive age women in Ethiopia (n = 1236)

Variables	Model 0	Model 1 AOR (95% CI)	Model 2 AOR (95% CI)	Model 3 AOR (95% CI)
**Individual level Characteristics**				
**Age**				
15–24		1		1
25–34		0.25 (0.12, 0.53)		2.6 (1.2, 5.5)*
35–49		0.15 (0.06, 0.33)		1.5 (1.06,3.3)*
**Educational status of** **the respondents**				
No formal education		1		1
Primary education		2.03 (1.14, 3.6)		2.25 (0.98, 4.33)
Secondary and above		3.0 (2.19, 8.82)		3.41 (2.19, 4.88)*
**Marital status**				
Married		0.36 (0.17, 0 0.75)		0.42 (0.22, 1 0.77)
Not married		1		1
**Media exposure**				
Yes		3.51 (1.96, 6.29)		2.97 (1.56, 5.64)*
No		1		1
**Wealth index**				
Poor		1		1
Middle		0.91 (0.40, 2.07)		0.99 (0.43, 2.24)
Rich **Religion**		1.19 (0.57, 2.48)		1.29 (0.62, 2.70)
Orthodox Christian		1		1
Muslim		1.69 (1.05, 2.70)		0.32 (0.15, 0.66)
Protestant		2.20 (1.01, 4.85)		1.04 (0.50, 2.17)
Others+		2.20 (1.01, 4.85)		2.84 (0.44, 18.04)
**Community level variables**				
**Residency**				
Urban		2.0 (1.2, 5.00)		3.19 (1.20, 5.23)*
Rural		1		1
**Community level education**				
High			0.41 (0.22, 0.79)	1.35 (0.61, 2.96)
Low			1	1
**Community media exposure**				
Low			1	1
High			3.49 (0.33, 5.63)	1.25 (0.55, 2.82)
**Region**				
Metropolitan			2.05 (1.31, 3.24)	2. 68 (1.46, 4.74)*
Large central			0.88 (0.28, 2.81)	1.29 (0.40, 4.11)
Small peripheral			1	1

* Statistically significant, AOR Adjusted Odds Ratio, COR Crude Odds Ratio

Null model: adjusted for individual-level characteristics, Model 2: Adjusted for community-level characteristics, Model 3: adjusted for both individual and community level characteristics

+Others = Catholic, traditional and other EDHS category

## Discussion

The actual implication of inadequate emergency contraception knowledge is regarded as one of the primary and leading causes of induced abortion, spontaneous abortion, or stillbirth, because the majority of post-abortion women are practically immediately at risk for pregnancy. Therefore, this analysis had revealed knowledge of the emergency contraceptive and its associated factors among abortion experienced reproductive women in Ethiopia. A total of 1236 reproductive age women were included in the analysis and only 17.19% (95% CI: 15.18, 19.40) were found to be knowledgeable about emergency contraceptives. This study was lower than a study conducted in Drie Dawa (34.1%) [7], Tigray (40.4%) [36], Jima University (65.7%) [37], south Ethiopia (72.2%) [38], south eastern Nigeria (51.6%) [22], Tamale Ghana (69%) [39]. However, this finding is higher in study conducted in Bangladesh (14%) [40]. This difference can result from the study’s multilevel methodology or from sociocultural differences with respect to other nations. At the same time variations could result from differences in sample size, study design, and awareness of contraceptive options among different nations.

Knowledge of EC remained higher for the respondents aged 25 to 34 and 35 to 49 compared to their younger with (AOR = 2.6; 95% CI: 1.2, 5.5), (AOR = 1.5; 95% CI: 1.06, 3.3) 15 to 24 aged women respectively. This outcome was consistent with research from Deberemarkos, Adama, and Mekele Universities [41–43]. The rationale could be that as people get older, they are likely exposed to more information regarding emergency contraception.

The likelihood of EC knowledge increased as the level of education of the study participants increased. Those respondents with secondary & above were more likely to have EC knowledge compared to no formally educated women. This can be explained by the notion that women with higher levels of education have better access to health care information. Education has a favorable impact on women’s ability to comprehend reproductive health issues and choose the optimal contraceptive technique for their individual health needs. Also, it raises women’s status generally in terms of their knowledge, attitudes, and health-seeking behavior [44, 45].

Women who exposed to media were positively associated with emergency contraceptive knowledge. This result is in line with a study conducted in Dire Dawa [7]. A possible explanation is that the media has a powerful ability to explain different methods, their benefits and where they are available to women, enhancing women’s knowledge of the emergency contraceptives.

This study revealed that women residing in urban areas had a higher knowledge of EC than that of rural areas. Similarly, a study in developing countries also revealed higher knowledge of EC in urban areas [46]. This can be explained by factors including low socioeconomic position, limited access to information, and poorer educational attainment in rural locations. In addition, in most regions, women in urban areas have better access to the Internet, media outlets, and healthcare providers than women in rural regions [47–49]. Many other reproductive health outcomes, like EC, were also better in cities [50, 51].

Moreover, women living in metropolitan region were 2. 68 times more likely to have knowledge of emergency contraceptive compared to women in the small peripheral regions. This demonstrates that there was regional heterogeneity of knowledge of EC in Ethiopia, which was also found in another study [52].

The most recent nationally representative data included in this investigation were gathered using standardized and established data gathering methods. Additionally, multilevel analysis (an advanced model) was used to account for the linked nature of the EDHS data in the estimation process. Despite the foregoing benefits, the study’s cross-sectional design prevents it from demonstrating the causal link between the outcome and the independent factors.

## Conclusion

Emergency contraceptive knowledge among abortion experienced reproductive age women in Ethiopia was low. Women age, educational status of mothers, media exposure, residency, and region were significantly associated with emergency contraceptive knowledge. The lower degree of EC knowledge indicates the need to take into account potential FP service structure modifications while formulating various policy-level initiatives. To enhance understanding and use of ECs in the current Ethiopian setting, it is imperative to ensure exposure to EC information, particularly in rural regions.

## Data Availability

Data for this study were sourced from Ethiopian Demographic and Health surveys (EDHS), which is freely available online at (https://dhsprogram.com).

## References

[1] Tesfaye K (2019). Assessment of Knowledge, attitude, practice and Associated factors on emergency Contraception among Level-Ii to Level-Iv Female students in Hawassa College of Health Sciences, South, Ethiopia. J Med Care Res Rev.

[2] Gupta RK, Singh P, Gupta C, Kumari R, Langer B, Gupta R (2017). Emergency contraception: knowledge, attitude and practices among recently married females in a rural area of North India. Int J Res Med Sci.

[3] Schwarz EB, Gerbert B, Gonzales R (2007). Need for emergency contraception in urgent care settings. Contraception.

[4] Ottesen S, Narring F, Renteria S-C, Michaud P-A (2002). Emergency contraception among teenagers in Switzerland: a cross-sectional survey on the sexuality of 16-to 20-year-olds. J Adolesc Health.

[5] Adhikari R (2009). Factors affecting awareness of emergency contraception among college students in Kathmandu, Nepal. BMC Womens Health.

[6] Harper CC, Speidel JJ, Drey EA, Trussell J, Blum M, Darney PD (2012). Copper intrauterine device for emergency contraception: clinical practice among contraceptive providers. Obstet Gynecol.

[7] Abate M, Assefa N, Alemayehu T (2014). Knowledge, attitude, practice, and determinants emergency contraceptive use among women seeking abortion services in Dire Dawa, Ethiopia. PLoS ONE.

[8] Tamire W, Enqueselassie F (2007). Knowledge, attitude, and practice on emergency contraceptives among female university students in Addis Ababa, Ethiopia. Ethiop J Health Dev.

[9] Merchant RC, Casadei K, Gee EM, Bock BC, Becker BM, Clark MA (2007). Patients’ emergency contraception comprehension, usage, and view of the emergency department role for emergency contraception. J Emerg Med.

[10] Myer L, Mlobeli R, Cooper D, Smit J, Morroni C (2007). Knowledge and use of emergency contraception among women in the Western Cape province of South Africa: a cross-sectional study. BMC Womens Health.

[11] Babaee G, Jamali B, Mirmohammad Ali M (2003). Investigating the knowledge, attitude and its relationship with the mean of using emergency contraception. J Sex &Marital Therapy.

[12] Seife M, Fikre E. Assessment of level of awareness and utilization of emergency contraception, among college female students in Oromia Regional state, Arsi Zone, Asella. *South-East Ethiopia, Mater Thesis Addis Ababa University* 2007.

[13] Sedgh G, Singh S, Hussain R (2014). Intended and unintended pregnancies worldwide in 2012 and recent trends. Stud Fam Plann.

[14] Singh S, Sedgh G, Hussain R (2010). Unintended pregnancy: worldwide levels, trends, and outcomes. Stud Fam Plann.

[15] Organization WH. Facts on induced abortion worldwide. *World Health Organization, Geneva* http://www.whoint/reproductivehealth/publications/unsafe_abortion/induced_abortion_2012pdf 2012

[16] Singh S, Fetters T, Gebreselassie H, Abdella A, Gebrehiwot Y, Kumbi S, Audam S. The estimated incidence of induced abortion in Ethiopia, 2008. Int Perspect Sex Reproductive Health 2010:16–25. 10.1363/ipsrh.36.016.10 20403802

[17] Gebreselassie H, Fetters T, Singh S, Abdella A, Gebrehiwot Y, Tesfaye S, Geressu T, Kumbi S. Caring for women with abortion Complications in Ethiopia: national estimates and future implications. Int Perspect Sex Reproductive Health 2010:6–15. 10.1363/ipsrh.36.006.10 20403801

[18] Mesfin D (2020). Emergency contraceptive knowledge, utilization and associated factors among secondary school students in Wolkite town, southern Ethiopia, cross sectional study. Contracept Reproductive Med.

[19] Tajure N. Knowledge, attitude and practice of emergency contraception among graduating female students of Jimma University, Southwest Ethiopia. Ethiop J Health Sci 2010, 20(2). PMC3275837 22434966

[20] Busery S, Sisay M (2016). Knowledge, attitude and practice of emergency contraceptives among graduating female students of college of health and medical sciences, Haramaya University, Eastern Ethiopia. Sch Acad J Pharm.

[21] Shiferaw BZ, Gashaw BT, Tesso FY (2016). Knowledge, attitude and practice of emergency contraceptives among Mizan-Tepi university female students, South West Ethiopia. J Pain Manage Med.

[22] Ezebialu I, Eke A (2013). Knowledge and practice of emergency contraception among female undergraduates in south eastern Nigeria. Annals of Medical and Health Sciences Research.

[23] Hoque ME, Ghuman S. Knowledge, practices, and attitudes of emergency contraception among female university students in KwaZulu-Natal, South Africa. 2012. 10.1371/journal.pone.0046346 PMC3458816 23050018

[24] Mutie I, Odero M, Mbugua G (2012). The prevalence and Knowledge of Emergency Contraceptive Pills (ECP’s) among women in Kibera, Nairobi. Afr J Health Sci.

[25] Kgosiemang B, Blitz J (2018). Emergency contraceptive knowledge, attitudes and practices among female students at the University of Botswana: a descriptive survey. Afr J Prim Health Care Family Med.

[26] Abate MG, Tareke AA (2019). Individual and community level associates of contraceptive use in Ethiopia: a multilevel mixed effects analysis. Archives of Public Health.

[27] Corsi DJ, Neuman M, Finlay JE, Subramanian S (2012). Demographic and health surveys: a profile. Int J Epidemiol.

[28] Csa I. Central statistical agency (CSA)[Ethiopia] and ICF. *Ethiopia demographic and health survey, Addis Ababa, Ethiopia and Calverton, Maryland, USA* 2016, 1.

[29] Liyew AM, Teshale AB (2020). Individual and community level factors associated with anemia among lactating mothers in Ethiopia using data from Ethiopian demographic and health survey, 2016; a multilevel analysis. BMC Public Health.

[30] Getaneh T, Negesse A, Dessie G, Desta M, Moltot T (2020). Predictors of unmet need for family planning in Ethiopia 2019: a systematic review and meta analysis. Archives of Public Health.

[31] Shifti DM, Chojenta C, Holliday G, Loxton E (2020). Individual and community level determinants of short birth interval in Ethiopia: a multilevel analysis. PLoS ONE.

[32] Belachew TB, Asmamaw DB, Negash WD (2023). Short birth interval and its predictors among reproductive age women in high fertility countries in sub-saharan Africa: a multilevel analysis of recent demographic and health surveys. BMC Pregnancy Childbirth.

[33] Kefale B, Yalew M, Damtie Y, Adane B. A multilevel analysis of factors associated with teenage pregnancy in Ethiopia. Int J women’s health 2020:785–93. 10.2147/IJWH.S265201 PMC7548018 33116928

[34] Shagaro SS, Gebabo TF, Mulugeta BT (2022). Four out of ten married women utilized modern contraceptive method in Ethiopia: a multilevel analysis of the 2019 Ethiopia mini demographic and health survey. PLoS ONE.

[35] Birhanu BE, Kebede DL, Kahsay AB, Belachew AB (2019). Predictors of teenage pregnancy in Ethiopia: a multilevel analysis. BMC Public Health.

[36] Abraha D, Welu G, Berwo M, Gebretsadik M, Tsegay T, Gebreheat G, Gebremariam H. Knowledge of and Utilization of Emergency Contraceptive and Its Associated Factors among Women Seeking Induced Abortion in Public Hospitals, Eastern Tigray, Ethiopia, 2017: A Cross-Sectional Study. *BioMed Research International* 2019, 2019. 10.1155/2019/7209274 PMC6886317 31828125

[37] Wodaynew T, Bekele D (2021). Assessment of knowledge, attitude and practice of contraceptive use among postpartum women in Jimma University medical center, Jimma Town, South West Ethiopia. Int J Womens Health Wellness.

[38] Tolossa E, Meshesha B, Abajobir AA. Assessment of level of knowledge and utilization of emergency contraception among female students of Hawassa University, South Ethiopia. *Advances in reproductive sciences* 2013, 2013.

[39] Amalba A, Mogre V, Appiah MN, Mumuni WA (2014). Awareness, use and associated factors of emergency contraceptive pills among women of reproductive age (15–49 years) in Tamale, Ghana. BMC Womens Health.

[40] Alam MZ, Islam MS, Sultan S (2020). Knowledge and practice of emergency contraception among currently-married women in Bangladesh: evidence from a national cross-sectional survey. J Popul Social Stud [JPSS].

[41] Tessema M, Bayu H (2015). Knowledge, attitude and practice on emergency contraception and associated factors among female students of Debre-Markos University, Debre-Markos Town, East Gojam Zone, North West Ethiopia, 2013. Glob J Med Res.

[42] Tilahun D, Assefa T, Belachew T. Knowledge, attitude and practice of emergency contraceptives among Adama University female students, Ethiopia. Ethiop J Health Sci 2010, 20(3). 10.4314/ejhs.v20i3.69449 PMC3275848 22434979

[43] Abera H, Mokonnen M, Jara D (2014). Knowledge, attitude, Utilization of Emergency Contraceptive and Associated Factors among female students of Debre Markos Higher Institutions, Northwest Ethiopia, 2014. Fam Med Med Sci Res.

[44] Alemayehu GA, Fekadu A, Yitayal M, Kebede Y, Abebe SM, Ayele TA, Gizaw Z, Wubeshet M, Muchie KF, Gelagay AA (2018). Prevalence and determinants of contraceptive utilization among married women at Dabat Health and demographic Surveillance System site, northwest Ethiopia. BMC Womens Health.

[45] Oumer M, Manaye A, Mengistu Z. Modern contraceptive method utilization and associated factors among women of reproductive age in Gondar City, Northwest Ethiopia. Open Access Journal of Contraception 2020:53–67. 10.2147/OAJC.S252970 PMC7322113 32612400

[46] Tesfa A, Bizuneh D, Tesfaye T, Gebru AA, Ayene YY, Tamene A (2015). Assessment of knowledge, attitude and practice towards emergency contraceptive methods among female students in Seto Semero high school, Jimma town, South West Ethiopia. Sci J Public Health.

[47] Marron O, Thomas G, Burdon Bailey JL, Mayer D, Grossman PO, Lohr F, Gibson AD, Gamble L, Chikungwa P, Chulu J (2020). Factors associated with mobile phone ownership and potential use for rabies vaccination campaigns in southern Malawi. Infect Dis Poverty.

[48] Duggan M, Brenner J. The demographics of social media users, 2012, vol. 14: Pew Research Center’s Internet & American Life Project Washington, DC; 2013.

[49] Owolabi OO, Wong KL, Dennis ML, Radovich E, Cavallaro FL, Lynch CA, Fatusi A, Sombie I, Benova L (2017). Comparing the use and content of antenatal care in adolescent and older first-time mothers in 13 countries of West Africa: a cross-sectional analysis of demographic and health surveys. The Lancet Child & Adolescent Health.

[50] Alam MZ, Sultan S (2019). Knowledge and practice of menstrual regulation (MR) in Bangladesh: patterns and determinants. J Popul Social Stud [JPSS].

[51] Khan SH, Talukder SH (2013). Nutrition transition in B angladesh: is the country ready for this double burden. Obes Rev.

[52] Benevides R, Fikree F, Holt K, Forrester H. Four country case studies on the introduction and scale-up of emergency contraception. *Evidence to Action Project, Washington, DC* 2014.

